# First Evaluation of Glucose-6-Phosphate Dehydrogenase (G6PD) Deficiency in Vivax Malaria Endemic Regions in the Republic of Korea

**DOI:** 10.1371/journal.pone.0097390

**Published:** 2014-05-22

**Authors:** Youn-Kyoung Goo, So-Young Ji, Hyun-Il Shin, Jun-Hye Moon, Shin-Hyung Cho, Won-Ja Lee, Jung-Yeon Kim

**Affiliations:** 1 Division of Malaria and Parasitic Diseases, National Institute of Health, Korea CDC, Osong Saeng-myeong 2 ro, Osong Health Technology Administration, Osong, Republic of Korea; 2 Department of Parasitology and Tropical Medicine, Kyungpook National University School of Medicine, Daegu, Republic of Korea; Agency for Science, Technology and Research - Singapore Immunology Network, Singapore

## Abstract

**Background:**

Glucose-6-phosphate dehydrogenase (G6PD) deficiency is the most common human enzyme defect and affects more than 400 million people worldwide. This deficiency is believed to protect against malaria because its global distribution is similar. However, this genetic disorder may be associated with potential hemolytic anemia after treatment with anti-malarials, primaquine or other 8-aminoquinolines. Although primaquine is used for malaria prevention, no study has previously investigated the prevalence of G6PD variants and G6PD deficiency in the Republic of Korea (ROK).

**Methods:**

Two commercialized test kits (Trinity G-6-PDH and CareStart G6PD test) were used for G6PD deficiency screening. The seven common G6PD variants were investigated by DiaPlexC kit in blood samples obtained living in vivax malaria endemic regions in the ROK.

**Results:**

Of 1,044 blood samples tested using the CareStart G6PD test, none were positive for G6PD deficiency. However, a slightly elevated level of G6PD activity was observed in 14 of 1,031 samples tested with the Trinity G-6-PDH test. Forty-nine of the 298 samples with non-specific amplification by DiaPlexC kit were confirmed by sequencing to be negative for the G6PD variants.

**Conclusions:**

No G6PD deficiency was observed using phenotypic- or genetic-based tests in individuals residing in vivax malaria endemic regions in the ROK. Because massive chemoprophylaxis using primaquine has been performed in the ROK military to kill hypnozoites responsible for relapse and latent stage vivax malaria, further regular monitoring is essential for the safe administration of primaquine.

## Introduction

Malaria is a devastating protozoan disease that afflicts several hundred million people annually. Vivax malaria, which is caused by *Plasmodium vivax*, is the most widespread human malaria and is endemic to the tropical and subtropical countries of the Americas and Asia, including the Republic of Korea (ROK) [1], [2]. Unlike *P. falciparum*, *P. vivax* is characterized by hypnozoites in the liver, which initiate secondary blood-stage infection [3]. Thus, to eliminate vivax malaria, successful strategies must reduce the clinical burden and completely deplete the parasite reservoir by killing silent hypnozoites. Primaquine is the only effective drug for both preventive prophylaxis as well as for killing *P. vivax* liver hypnozoites. For this reason, primaquine has been administered to soldiers serving in endemic regions in the ROK.

Glucose-6-phosphate dehydrogenase (G6PD) is an X-linked essential enzyme that protects cells from oxidative stress, particularly red blood cells. G6PD deficiency, the most common known enzymopathy, is a hereditary genetic defect. It is one of the most prevalent polymorphisms in humans, particularly in males. Since the global distribution of G6PD-deficient variants resembles the geographical distribution of malaria, it has been postulated that G6PD deficiency confers some protection against malaria. Furthermore, a number of G6PD deficient variants have been reported in sub-Saharan Africa and Southeast Asia [4]–[6]. G6PD deficiency is associated with several clinical disorders such as neonatal jaundice, favism, and hemolytic anemia after certain infections and medications [7].

G6PD deficiency is of interest because primaquine-induced hemolysis increases in the presence of G6PD variants. 8-aminoquinolines, including primaquine, are administered to patients infected with *P. vivax* to prevent relapse. They are also administered to patients infected with *P. falciparum* to reduce gametocyte carriage to block transmission. However, primaquine can cause intravascular hemolysis in individuals with G6PD deficiency, which, depending on the G6PD variant, may increase the risk of primaquine-induced hemolysis from mild to severe [8]. Three known primaquine-sensitive phenotypes, African A-, Mahidol, and Mediterranean B-, result in mild, moderate, and severe hemolysis, respectively. Although several factors such as total primaquine dose, patient age, and G6PD variant type influence risk of death, investigators calculated the risk of death associated with primaquine ingestion at approximately 1 in 692,307 [9]. Thus, G6PD deficiency testing and a G6PD genotyping are required before treatment.

In the ROK, vivax malaria endemic regions are concentrated near the demilitarized zone (DMZ), and thus, military personnel and residents living in the area are at risk of contracting vivax malaria. Since 1997, chloroquine and primaquine chemoprophylaxis has been administered to soldiers without any G6PD deficiency testing. To date, no study has been undertaken to ascertain the prevalence of G6PD deficiency in the ROK. This study evaluated G6PD enzymatic activity and the prevalence of G6PD variants in individuals living in the vivax malaria endemic Yeon-cheon and Pa-ju regions of the ROK.

## Materials and Methods

### Ethics statements

The study was approved by the ethics committee of the Korea National Institute of Health. For each participant, a written consent form was used to obtain informed consent and permission for the collection of a 5 ml blood sample.

### Samples

**Figure 1:**
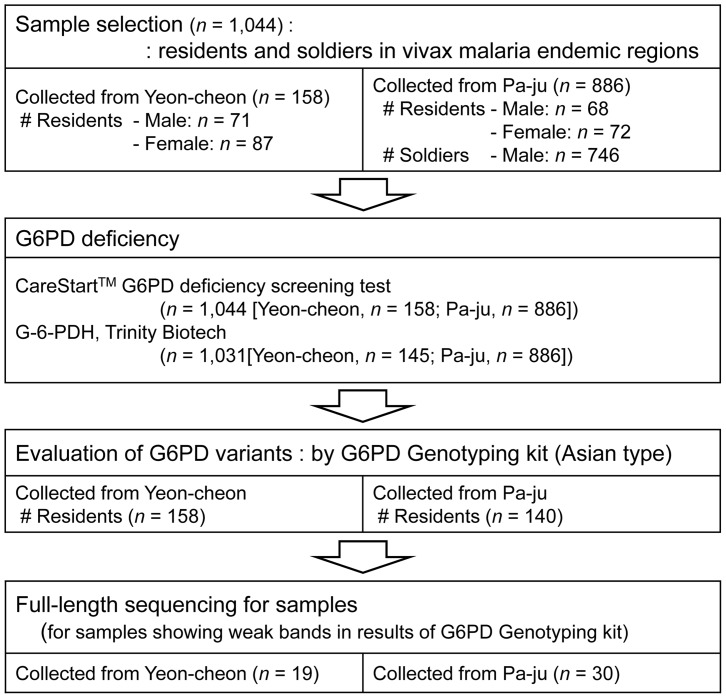
Sample selection profile for the evaluation of G6PD enzyme activity and G6PD variants.

**Figure 2:**
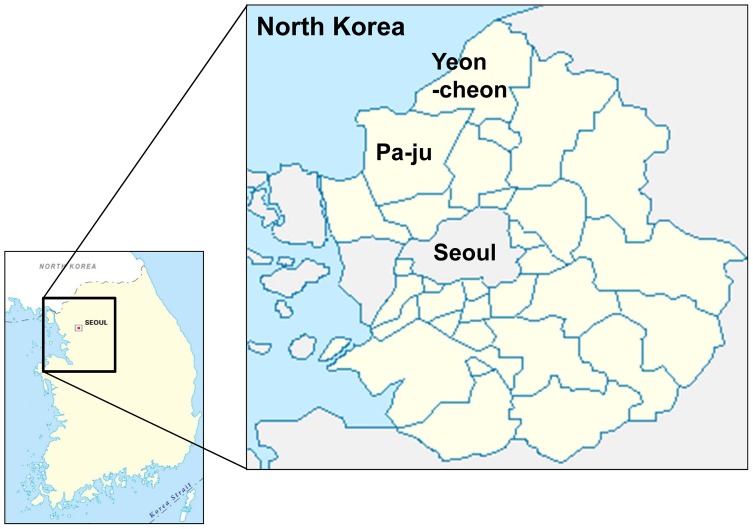
Map of Yeon-cheon and Pa-ju: endemic regions of vivax malaria in ROK in which samples were collected.

This study was conducted at the Division of Malaria and Parasitic Diseases of the Korea Centers for Disease Control and Prevention (KCDC) from February 2012 to February 2013. EDTA preserved venous blood samples were obtained from 1,044 individuals (residents (*n* = 298) and soldiers (*n* = 746)), living in the endemic area (Yeon-cheon and Pa-ju; Figure 1 and 2). The tested individuals consist of 159 females (15.2%) and 885 males (84.8%). Nested-PCR was used to confirm a diagnosis of vivax malaria, and all samples were negative [10].

### Determination of G6PD activity

Two commercial kits, the CareStart G6PD deficiency screening kit (AccessBio, USA) and the Trinity G-6-PDH kit (Trinity Biotech, Ireland), were used to screen for G6PD deficiency. The CareStart G6PD deficiency screening test is a qualitative enzyme chromatographic test in rapid diagnostic test (RDT)-format. In accordance with the manufacturer's instructions, two drops of buffer were added to 2 µl of each of the 1,044 blood samples (298 residents and 746 soldiers) in a 96-well plate in duplicate. Test results were read visually after 10 mins. Samples with normal G6PD activity produced a distinct purple color background, whereas no color change was observed for G6PD deficient samples. Samples that produced a pale purple color were classified as normal. The 1,031 samples (285 residents and 746 soldiers) received in fresh condition within a maximum of 48 hours after sample collection were quantitatively tested for G6PD activity using the Trinity G-6-PDH test kit. All tests were conducted following the manufacturer's instructions [11]. The reliability of test results was monitored by calibration and by including three different controls provided by Trinity Biotech (deficient level: reference G5888; intermediate level: reference G5029; and normal level: reference G6888) in each test run. G6PD activities were expressed as units per gram of hemoglobin (U/g Hb). Test values ranging from 4.6 to 13.5 U/g Hb were considered to indicate normal G6PD activity. While conducting the Trinity G-6-PDH assay, the investigator and laboratory technicians were blinded to the CareStart test results.

### Analysis of G6PD variants

G6PD variants were detected using DiaPlexC G6PD Genotyping Kit (Asian type; SolGent, ROK), which enables the seven different variants of the G6PD gene to be screened by one-step PCR. The seven variants produce PCR products of different sizes, as follows; Vanua Lava (383 T>C, 154 bp), Mahidol (487 G>A, 337 bp), Coimbra (592 C>T, 234 bp), Viangchan (871 G>A, 501 bp), Union (1360 C>T, 803 bp), Canton (1376 G>T, 681 bp), and Kalping (1388 G>A, 557 bp). After genomic DNA extraction using the Genomic DNA Prep Kit (Blood) (SolGent, ROK) from the 298 blood samples obtained from residents, PCR mixtures were prepared using the DiaPlexC kit, and 2 µl of total genomic DNA (25–50 ng or 100 ng) was added to 23 µl of a master mixture containing premix (12.5 µl), G6PD (2 µl; Asian type) primer mixture, and distilled water (8.5 µl). The PCR cycling conditions used were as follows: initial denaturation at 95°C for 15 min, 30 amplification cycles (denaturation at 95°C for 30 sec, annealing at 60°C for 30 sec), and a final extension at 72°C for 40 sec. The PCR products were resolved by electrophoresis on 1% agarose gel, stained with safePinky (GenDEPOT, USA), and visualized under ultraviolet illumination. Each PCR reaction was confirmed by an internal control.

### G6PD amplification and G6PD variant sequencing

**Figure 3:**
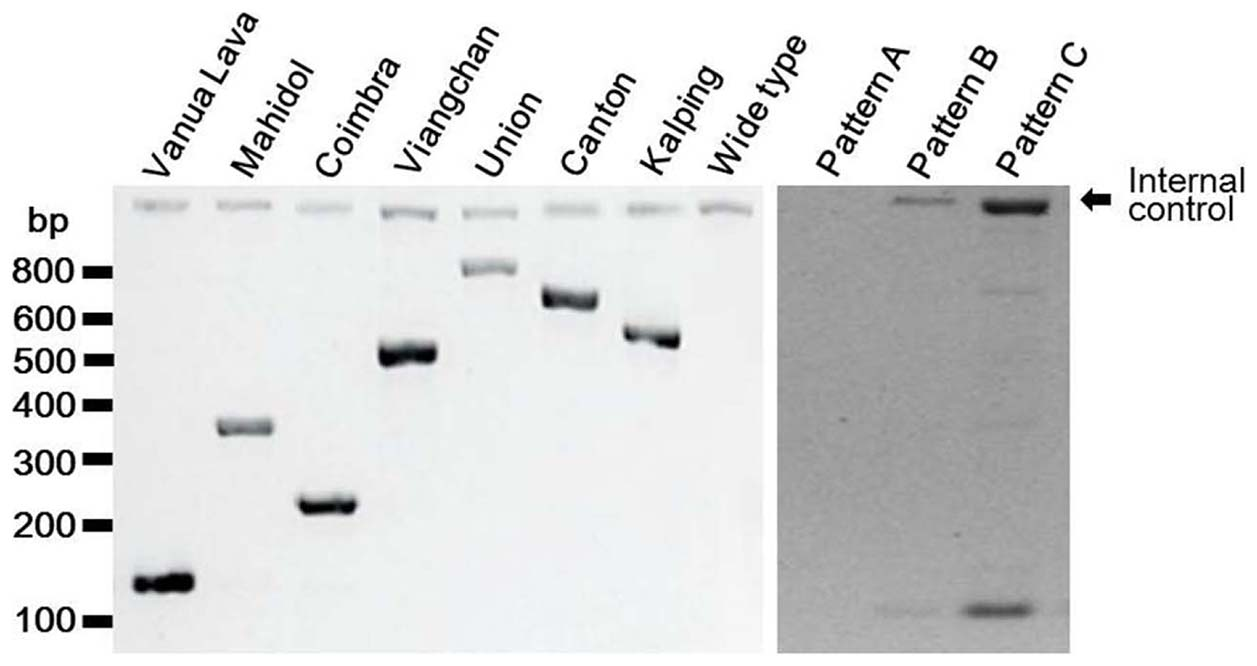
Results of screening for G6PD variants using the G6PD genotyping kit (Asian type, DiaPlexC) with G6PD variant controls (Vanua Lava, Mahidol, Coimbra, Viangchan, Union, Canton, and Kalping mutants) and the three patterns produced by the DiaPlexC test.

**Figure 4:**
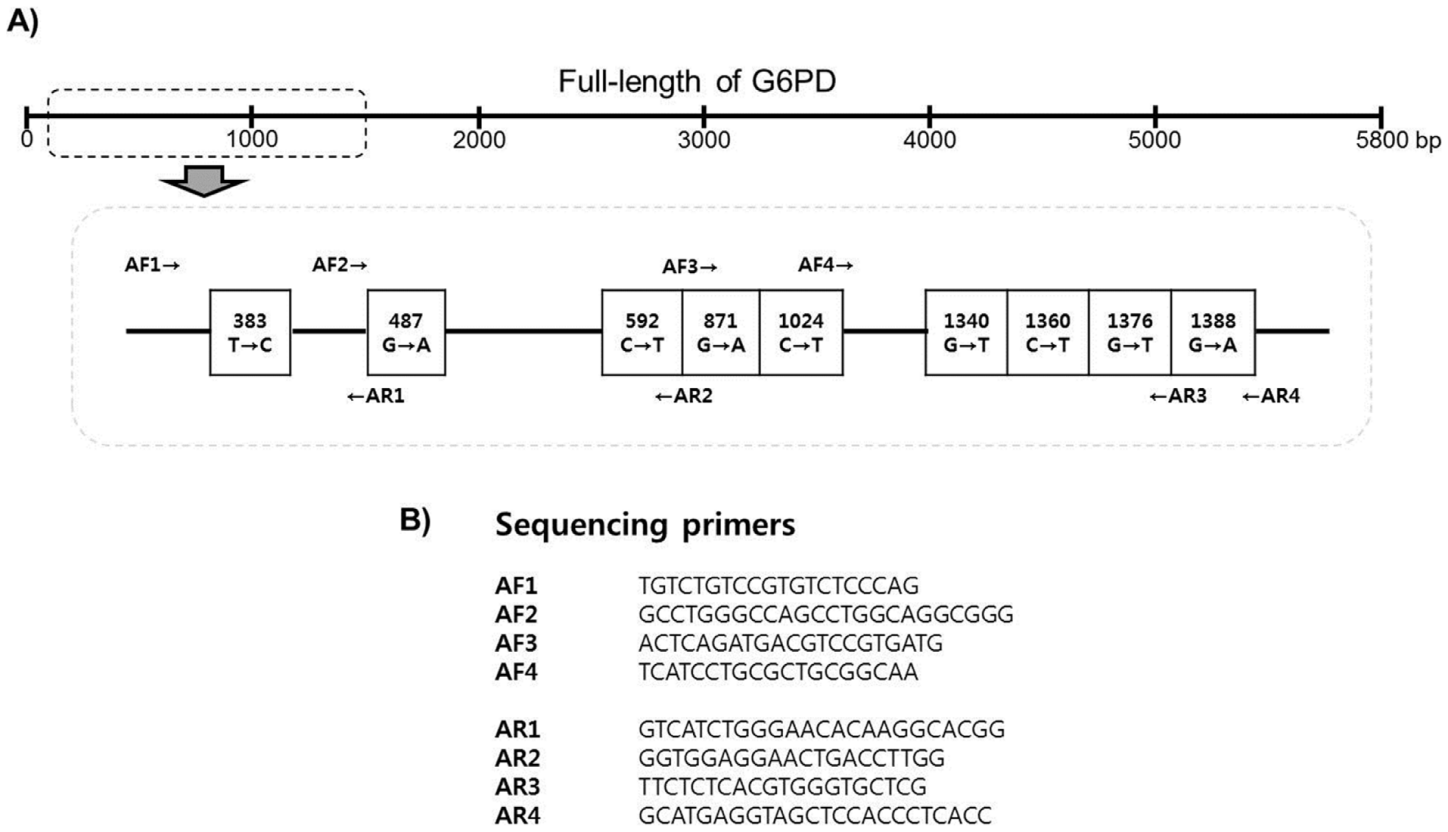
Schematic diagram of G6PD variants (A) and a list of the primers used for G6PD variant sequencing (B).

Forty-nine blood samples (19 samples from Yeon-cheon and 30 samples from Pa-ju) with multiple bands (Figure 3) were selected for analysis, and all G6PD variants were analyzed by sequencing. For the sequencing analysis, full-length (5.8 kb) G6PD genes were amplified from genomic DNA and purified using the QIAquick PCR purification kit (Qiagen, USA). DNA sequencing was performed for nine variant sites using the primers shown in Figure 4: the Chinese 5 (1024 C>T) and Jiangxi (1340 G>T) variants and the seven above-mentioned variants.

## Results and Discussion

Phenotypic (biochemical) and genetic techniques are currently available to determine G6PD activity status. Phenotypic tests are the most widely used and are the best indicators of G6PD function levels. Some commercialized G6PD phenotype deficiency-testing products are also suitable for point-of-care use in clinics because they are rapid and straightforward. DNA-based genotyping could also be applied to large-scale screening programs to provide potentially quantitative measures and details of geographical deficiencies. In present study, phenotypic and DNA-based genotypic testing was conducted to assess the prevalence of G6PD deficiency in the ROK.

A total of 1,044 blood samples were collected in two cities, Yeon-cheon (*n = *158) and Pa-ju (*n = *886), in which vivax malaria is endemic. Simultaneously with blood collection, hemoglobin levels were measured to determine whether subjects were anemic. Higher mean hemoglobin levels were measured in the Yeon-cheon group (14.5 g/dL *vs*. 13.6 g/dL). Additionally, mean hemoglobin levels were higher in males than in females (14.0 g/dL *vs*. 13.4 g/dL), which is consistent with findings reported elsewhere [12]. Twelve subjects from Yeon-cheon and 40 from Pa-ju had a medical history of vivax malaria infection, and those from Yeon-cheon had been more recently infected (mean 8.2 years *vs*. 12.0 years).

To screen for G6PD enzyme activity, the Trinity G-6-PDH kit and the CareStart G6PD deficiency screening kit were used to test 1,031 (residents (*n = *285) and soldiers (*n = *746)) and 1,044 (residents (*n = *298) and soldiers (*n = *746)) blood samples, respectively. The Trinity G-6-PDH kit is suitable for the quantitative measurement of G6PD enzyme activity based on enzyme kinetics. Although this kit is most commonly used, it is labor-intensive and time consuming. The CareStart G6PD deficiency screening kit provides a rapid diagnostic test result and is an attractive alternative for G6PD enzyme assays. The CareStart kit detects moderate and severe enzyme deficiencies (<2.7 U/g Hb), but levels of <2 U/g Hb are not detected [12]. All 1,044 tested samples showed normal G6PD activity according to the CareStart G6PD assays. Next, for the Trinity G-6-PDH kit assay, the standard value, presenting normal G6PD enzyme activity, of the Trinity G-6-PDH kit was calculated using three controls supplied with the kit. The normal range of G6PD enzyme activity values for the Trinity G-6-PDH kit was 4.6–13.5 U/g Hb, and this was defined as the standard range for G6PD enzymatic activity. Interestingly, in contrast to a previous Cambodian study [12], in which values of the Trinity G-6-PDH assay were significantly higher for males than females, the results of our 1,031 samples showed similar levels. Fourteen samples had slightly elevated values with the Trinity G-6-PDH kit (range 13.60-13.87 U/g Hb, normal range 4.6-13.5 U/g Hb) (Table 1). These 14 individuals (4 females and 10 males) had no history of vivax malaria. Since slightly higher values do not reflect G6PD deficiency, these results could be considered normal. In addition, no participant from Yeon-cheon or Pa-ju had a low G6PD enzyme activity (<4.6 g/U Hb), which would suggest a low prevalence of G6PD deficiency in the ROK. However, given that a study in the ROK by Blackwell et al. found that less than 0.9% of Korean males had a G6PD deficiency, it is possible that the absence of findings in our sample was due to a small sample size (885 males) rather than a true absence of G6PD deficiency in our population of interest [13]. Therefore, continued subject recruitment is required to ascertain conclusively whether the findings reported here are accurate.

**Table 1:** Details of fourteen individuals with slightly elevated G6PD activity in the Trinity G-6-PD kit.

Sample No.	Region	Age	Gender	Trinity (U/g Hb) *	AccessBio
1	Yeon-cheon	46	Female	13.68	Negative
2	Yeon-cheon	79	Female	13.68	Negative
3	Yeon-cheon	ND	Female	13.68	Negative
4	Yeon-cheon	81	Male	13.7	Negative
5	Yeon-cheon	63	Female	13.7	Negative
6	Yeon-cheon	75	Male	13.78	Negative
7	Yeon-cheon	75	Male	13.78	Negative
8	Pa-ju	20–30**	Male	13.52	Negative
9	Pa-ju	20–30**	Male	13.54	Negative
10	Pa-ju	20–30**	Male	13.57	Negative
11	Pa-ju	20–30**	Male	13.61	Negative
12	Pa-ju	20–30**	Male	13.67	Negative
13	Pa-ju	20–30**	Male	13.71	Negative
14	Pa-ju	20–30**	Male	13.87	Negative

ND  =  No data.

*: Normal level of G6PD activity by Trinity kit  = 4.6–13.5 U/g.

**: Samples were collected from Korean soldiers aged 20–29 years.

In order to obtain more details on G6PD deficiency, a G6PD genotyping study was performed using the G6PD Genotyping Kit (Asian type; Solgent) using the 298 samples except samples from soldiers. The kit provides a simple and rapid alternative to PCR and enzyme digestion-based assays [14], [15], and is able to detect six different G6PD variants, Vanua Lava (383 T>C), Mahidol (487 G>A), Coimbra (592 C>T), Viangchan (871 G>A), Union (1360 C>T), Canton (1376 G>T), and Kalping (1388 G>A), by one-step PCR.

As shown in Figure 3, three different patterns of bands were observed. Forty-one samples (28 and 13 samples from Yeon-cheon and Pa-ju, respectively) showed pattern A, which exhibits a weak or absent internal control band. Target gene amplification for the 41 samples was repeated using a higher concentration of genomic DNA (25–50 ng vs. 100 ng); this resulted in the B pattern, which represents the wide type, for all 41 samples. Pattern C resulted from non-specific amplification. Forty-nine samples (19 and 30 samples from Yeon-cheon and Pa-ju, respectively) were found to have more than three bands, and were therefore suspected of containing G6PD variants. To determine whether these were non-specific bands or real G6PD variants, the full-length G6PD gene was amplified in those 49 samples and subsequently sequenced for each variant site. Sequencing analyses revealed that all 49 samples had wild type in all nine possible G6PD variants sites: Vanua Lava (383 T>C), Mahidol (487 G>A), Coimbra (592 C>T), Viangchan (871 G>A), Union (1360 C>T), Canton (1376 G>T), Kalping (1388 G>A), Chinese 5 (1024 C>T), and Jiangxi (1340 G>T). Although the manufacturer did not provide specificity and sensitivity information for this kit, we calculated the specificity at 83.6%. The prevalence and distribution of G6PD variants reportedly varies by geographic region, with Mahidol, Union, and Viangchan variant sites having been documented in Asian countries such as Thailand, Myanmar, Philippines, and China [15]–[19]. Furthermore, one geostatistical model-based mapping revealed that Asian countries are the centre of weight of G6PD deficiency-burdened populations [4]. It is interesting that no G6PD deficiency was detected in the ROK despite its proximity to countries with a strong presence of G6PD deficiency.

Malaria is considered as the single great natural selection criterion in malaria endemic regions [20]–[23]. This is supported by the fact that the geographical distribution of G6PD variants is consistent with malaria endemic areas. However, the effect of G6PD deficiency on *P. vivax* development is still controversial. Since vivax malaria re-emerged in the ROK in 1993, 29,692 cases of endemic vivax malaria have been reported between 1994 and 2010. Furthermore, approximately 20% (2,277 cases) of all vivax malaria cases (11,631 cases) reported between 2005 and 2013 were located in either the Yeon-cheon or Pa-ju region. On average, 253 cases of vivax malaria were occurred each year in the regions. The vivax malaria in the ROK has long latent periods followed by relapses that are caused by hypnozoites. To eliminate hypnozoites, massive primaquine chemoprophylaxis has been performed on military personnel stationed near the DMZ, although it is known that primaquine induces hemolysis in individuals with certain G6PD variants. More specifically, no treatment guidelines have been issued that account for this effect. In fact, high dose primaquine causes hemolysis in healthy individuals with G6PD wild type, and risk of hemolysis is very different in individuals with G6PD variants. For example, high dose primaquine cause only slight reductions in hemoglobin in those with the A-variant, but hemolysis of 25% of red blood cells occurs in those with the Mediterranean B-variant [24], [25]. In addition, weekly primaquine treatment for 8 weeks has been reported to be safer than a full 14-day course in the Mediterranean variant [26]. Thus, there is a need to optimize primaquine treatment with respect to safety and effectiveness and to develop a simple, accurate G6PD deficiency test to support clinical primaquine use. In present study, neither G6PD deficiency nor G6PD variant was detected in vivax malaria endemic regions in the ROK. It has been previously reported that G6PD deficiency is rare in the ROK, and a few cases of G6PD deficiency have been reported to date [27]. However, when it is considered that primaquine is administered routinely as chemoprophylaxis to military personnel in ROK, further regular screening of G6PD deficiency is needed to ensure the safe use of primaquine.

## Conclusions

Neither G6PD deficiency nor any G6PD variant was found in individuals residing in vivax malaria endemic regions in the ROK. This low incidence of G6PD deficiency is interesting because G6PD deficiency is prevalent in neighboring Asian countries. In view of the fact that primaquine chemoprophylaxis is performed routinely in military personnel stationed near the DMZ, regular screening for G6PD deficiency appears essential to ensure the safe administration of primaquine.
